# A social and news media benchmark dataset for topic modeling

**DOI:** 10.1016/j.dib.2022.108442

**Published:** 2022-07-04

**Authors:** Samuel Miles, Lixia Yao, Weilin Meng, Christopher M. Black, Zina Ben-Miled

**Affiliations:** a Department of Electrical and Computer Engineering, IUPUI, Indianapolis, IN 46202, USA; b Merck & Co., Inc., 90 E Scott Ave, Rahway, NJ 07065, USA; c Regenstrief Institute, Inc., 1101 W 10th Street, Indianapolis, IN 46202, USA

**Keywords:** Topic modeling, PSO, ETM, NVDM, Document embedding, Heath forums

## Abstract

Topic modeling is an active research area with several unanswered questions. The focus of recent research in this area is on the use of a vector embedding representation of the input text with both generative and evolutionary topic modeling techniques. Unfortunately, it is hard to compare different techniques when the underlying data and preprocessing steps that were used to develop the models are not available. This paper presents two secondary datasets that can help address this gap. These datasets are derived from two primary datasets. The first consists of 8145 posts from the r/Cancer health forum and the second consists of 18,294 messages submitted to 20 different news groups. The same preprocessing procedure is applied to both datasets by removing punctuation, stop words and high frequency words. Each dataset is then clustered using three different topic modeling techniques: pPSO, ETM and NVDM and three topic numbers: 10, 20, 30. In addition, for pPSO two text embeddings representation are considered: sBERT and Skipgram. The secondary datasets were originally developed in support of a comparative analysis of the aforementioned topic modeling techniques in a study titled “Comparing PSO-based Clustering over Contextual Vector Embeddings to Modern Topic Modeling” submitted to the Journal of Information Processing and Management. The present paper provides a detailed description of the two secondary datasets including the unique identifier that can be used to retrieve the original documents, the pre-processing scripts, the topic keywords generated by the three topic modeling techniques with varying topic numbers and embedding representations. As such, the datasets allow direct comparison with other topic modeling techniques. To further facilitate this process, the algorithm underlying the evolutionary topic modeling technique, pPSO, proposed by the authors is also provided.

## Specifications Table

**Table:** 

Subject	Artificial Intelligence
Specific subject area	Topic Modeling; Document Clustering
Type of data	Table
How the data were acquired	The raw documents are extracted from the r/Cancer [1] and the 20News-Groups [2] archives. The topic assignment generated by pPSO [3] are provided using the unique r/Cancer and 20NewsGroups document identifiers. The keywords for each topic generated by pPSO, ETM [4] and NVDM [5] are also included.
Data format	Analyzed
Description of data collection	The r/Cancer and 20News-Groups raw documents are retrieved from their respective archives [1,2]. Text pre-processing is then performed on the documents including removal of high frequency words, punctuation, numerical characters as well as forcing the text to lower case. Three topic modeling techniques (i.e., pPSO, ETM and NVDM) are applied to the pre-processed documents. Topic keywords are provided for each dataset and topic modeling technique with varying number of topics and embeddings when applicable.
Data source location	This paper describes a secondary dataset. The raw r/Cancer and 20News-Groups documents are obtained from the respective archives in [1,2]. They can be extracted using the provided unique document identifiers.
Data accessibility	The primary datasets can be retrieved from the r/Cancer [1] and 20News-Groups archives [2]. The secondary dataset is available from the following Repository: Repository name: A social and news media benchmark dataset for topic modeling Data identification number: 10.5281/Zenodo.6449720 Direct URL to data: https://doi.org/10.5281/Zenodo.6449720
Related research article	S. Miles, L. Yao, W. Meng, C.M. Black, Z. Ben Miled, Information Processing & Management. 59.3 (2022) 102,921. https://doi.org/10.1016/j.ipm.2022.102921

**Value of the Data**

• Public sharing of the fully labeled data rather than a subset of the data generated by the topic models allows the replication and validation of the results as well as enables direct comparison with competing topic modeling techniques.
• Topic modeling and health science researchers can derive benefit from this data.
• A well-defined benchmark from two different domains (i.e., online health forum and news groups) is an opportunity for a shared baseline for various NLP applications. It also allows for the exclusion of the text pre-processing techniques as a source of difference between various studies. The current benchmark can also be combined with other benchmarks, such as the one offered in [6], to a construct an extended dataset.
• The labeled documents can help promote retrospective studies that investigate topics important to the r/Cancer subscribers.

## 1 Data Description

The data release consists of two types of files: document and keyword files. The document file includes two columns: the document ID which uniquely identifies the raw document in the source archive, and the corresponding topic cluster ID assigned by pPSO. This file is only available for pPSO because this topic modeling technique is a hard classifier that assigns each document to a single topic. The other two techniques (i.e., ETM and NVDM) generate a probabilistic distribution of each document across all topics.

The second type of file is a keyword file which is provided for all topic modeling techniques. The file is organized by topic and includes the top ten topic keywords for each topic. An example keyword file is shown in Table 1.

**Table 1.** Structure of the keyword tables.

Topic_Num	Keyword 1	Keyword 2	Keyword 3	…
1	dads	hes	prostate	…
2	taste	food	meals	…
3	wigs	wig	shave	…
…	…	…	…	…

For each dataset and topic modeling technique, the number of topics was varied from 10, 20 to 30 topics. While the topics identified by each topic modeling technique for a given dataset may differ, the main themes are usually consistent. Table 2 shows some of the themes observed for the two datasets.

**Table 2.** Examples of observed topics for each dataset.

Dataset	Topic 1	Topic 2	Topic 3	Topic 4	Topic 5	…
2020 Reddit	foods	hair	female	male	treatment	…
20NewsGroups	sports	computer	space	religion	vehicle	…

## 2 Experimental Design, Materials and Methods

The protocol used to generate the topic models is provided under methods in the Repository. This protocol defines a data processing pipeline that is comprised of multiple steps:

• Data cleaning: This step includes the removal of stop words, high frequency words, and punctuation from raw documents. The text is also forced to lower case.
• Vector embedding: In this step, the initial embedding vector for each document produced in the previous step is generated from the pretrained language model sBERT [7]. This embedding is then reduced to a dimension of 300 using UMAP [8].
• Topic modeling: The source code for topic modeling using pPSO on the reduced embedded vectors allows for the clustering of the documents into distinct topics. The source code for ETM and NVDM can be found in [4,5], respectively.
• Data analysis: This is the final step in the pipeline. The topic keywords are extracted for each topic cluster and the topic coherence and diversity are computed.

## Ethics Statements

The raw r/Cancer data are extracted from a public domain archive [1]. Only the document identifiers are included in the secondary datasets. Users can retrieve the raw text data directly from the archive as per the Reddit redistribution and data sharing policies. The raw 20NewsGroups data is publicly available. However, we still follow the same procedure as in the r/Cancer dataset of only including the document identifier in the secondary dataset. No identified data is included in either secondary dataset.

## CRediT authorship contribution statement

**Samuel Miles:** Methodology, Software, Formal analysis, Investigation, Data curation, Writing – original draft. **Lixia Yao:** Conceptualization, Supervision. **Weilin Meng:** Conceptualization, Investigation, Supervision, Project administration. **Christopher M. Black:** Supervision, Project administration. **Zina Ben-Miled:** Conceptualization, Validation, Investigation, Writing – review & editing, Supervision.

## Declaration of Competing Interest

The authors declare that they have no known competing financial interests or personal relationships that could have appeared to influence the work reported in this paper.

## Data Availability

A Social and News Media Benchmark Dataset for Topic Modeling (Original data) (Zenodo).
